# Fenofibrate Protects Against Antipsychotic‐Induced Hyperglycemia Through Weight Loss and FGF21 Dependent Mechanisms

**DOI:** 10.1096/fj.202602558R

**Published:** 2026-09-08

**Authors:** Michael Akcan, Stewart Jeromson, Bradley J. Baranowski, Annalaura Bellucci, Serena Trang, Katelyn Eisner, Ailish Babicki‐Moore, Hadil Alfares, Meagan Arbeau, Kyle Medak, Margaret Hahn, David C. Wright

**Affiliations:** ^1^ School of Kinesiology University of British Columbia Vancouver British Columbia Canada; ^2^ BC Children's Hospital Research Institute Vancouver British Columbia Canada; ^3^ Schizophrenia Division Centre for Addiction and Mental Health Toronto Ontario Canada; ^4^ Institute of Medical Science University of Toronto Toronto Ontario Canada; ^5^ Department of Psychiatry University of Toronto Toronto Ontario Canada; ^6^ Banting & Best Diabetes Centre University of Toronto Toronto Ontario Canada; ^7^ Department of Pharmacology & Toxicology University of Toronto Toronto Ontario Canada; ^8^ Department of Clinical Medicine, Faculty of Health and Medical Sciences University of Copenhagen Copenhagen Denmark

**Keywords:** antipsychotics, dyslipidemia, FGF‐21, fibrates, hyperglycemia, metabolism, mice

## Abstract

Antipsychotic (AP) medications, such as olanzapine, cause acute weight gain independent of hyperglycemia through a glucagon‐dependent mechanism. Previous work has shown that exercise, a ketogenic diet, and fasting can protect against acute AP‐induced hyperglycemia and attenuate increases in glucagon. As these interventions all increase circulating concentrations of fibroblast growth factor 21 (FGF21), we hypothesized that increasing endogenous FGF21 through treatment with fenofibrate would protect against AP‐induced hyperglycemia. Male C57BL/6J mice were fed a low‐fat diet with or without fenofibrate (0.2% w/w) for 1 week prior to an acute olanzapine (OLZ) challenge. Fenofibrate reduced food intake and caused weight loss while protecting against OLZ‐induced increases in serum glucagon and blood glucose. Pair feeding mice with the same amount of food as fenofibrate‐treated animals conferred a similar degree of protection against OLZ‐induced hyperglycemia. Furthermore, an acute oral gavage with fenofibrate, despite increasing circulating FGF21 concentrations to a similar level as fenofibrate feeding, failed to protect against OLZ‐induced glucose excursions. The effects of fenofibrate on weight loss and protection against OLZ‐induced hyperglycemia were attenuated in *FGF21* ^−/−^ mice. Fenofibrate attenuated both OLZ‐induced hyperlipidemia and increases in whole‐body fatty acid oxidation, with the latter effects being dependent upon FGF21. Together, our findings demonstrate that fenofibrate protects against acute olanzapine‐induced hyperglycemia and hyperlipidemia in a weight loss and FGF21‐dependent manner.

## Graphical abstract

The proposed mechanism through which fenofibrate protects against olanzapine‐induced hyperglycemia in mice.

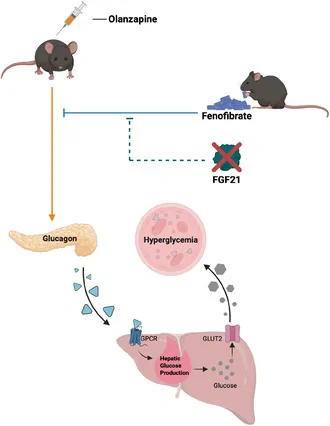

## 1 Introduction

Antipsychotics (APs) are the standard of care in the management of schizophrenia and a number of off‐label conditions such as anxiety, sleep disorders, dementia, bipolar disorder, and attention deficit disorder [1, 2, 3]. Driven primarily by off‐label usage, the prescription of APs has increased, with recent estimates suggesting a ∼60% increase globally between 2014 and 2019 [4]. Although effective at reducing psychoses, APs have serious long‐term metabolic complications including increases in the incidence of cardiovascular disease [5, 6] and obesity [7, 8]. While the chronic, long‐term complications of APs on metabolic health are thought to be largely driven by weight gain [7, 8, 9], there is an increasing appreciation of weight‐gain‐independent effects on glucose and lipid homeostasis. For instance, daily treatment with the AP olanzapine (OLZ) for three consecutive days, worsens glucose tolerance in human participants [10]. Similarly, fasting glucose concentrations are increased following a single dose of olanzapine in healthy human participants [11]. Weight‐gain‐independent effects of APs have been recapitulated in preclinical models. In this regard, work from our group [12, 13, 14, 15, 16, 17] and others [18, 19, 20] has demonstrated rapid impairments in glucose and lipid metabolism following acute OLZ treatment in C57BL/6 mice. Dyslipidemia is evident within hours of OLZ exposure, as evidenced by increases in serum fatty acids, hepatic fat content, and increased whole‐body fat oxidation [13, 14, 17]. Repeated bouts of hyperglycemia, as would occur following each dose of APs, can induce oxidative stress and endothelial dysfunction, which are contributors to the development of cardiometabolic disease [21, 22, 23].

Acute treatment with APs rapidly induces whole‐body insulin resistance [24, 25], increases liver glucose production [19], and causes a shift in fuel oxidation from carbohydrate to fat [13, 20]. A key mechanism mediating AP‐induced hyperglycemia involves glucagon. In this regard, we have shown that olanzapine treatment acutely increases serum glucagon in mice [17, 26, 27, 28, 29], an effect also shown in humans, where OLZ increases glucagon independent of weight gain [30, 31]. Importantly, we found that OLZ‐induced hyperglycemia and liver glucose production were absent in mice lacking the glucagon receptor [26].

Given the deleterious metabolic consequence of APs, there is growing interest in identifying strategies to mitigate these complications. Lifestyle interventions such as exercise [15, 17], fasting [32], and ketogenic diets [32] have been shown to protect against AP‐induced impairments in glucose metabolism. However, the implementation of these lifestyle approaches would be challenging in clinical populations given the negative symptoms of schizophrenia such as apathy, amotivation, and social withdrawal [31, 33]. This is further exemplified by the limited success of clinical trials implementing dietary and exercise interventions in participants with schizophrenia [34, 35]. Given the challenges of lifestyle modification, our group has been using lifestyle interventions as a tool to identify molecular pathways that could be targeted to counter the metabolic side effects of APs. One signaling molecule of particular interest is fibroblast growth factor 21 (FGF21), a peptide that increases in the circulation with fasting [36], ketogenic diets [37, 38], and exercise [39]. FGF21 is an atypical endocrine peptide hormone that is part of the FGF superfamily and is highly expressed in the liver [40], pancreas [41], and, to a lesser extent, in adipose tissue [42] and skeletal muscle [41]. FGF21 binds to its cognate FGF‐receptor and requires the co‐receptor β‐Klotho to form a stronger heterodimeric complex receptor [43, 44]. The restricted expression of β‐Klotho determines tissue selectivity for FGF‐21 action. Administration of FGF21 reduces circulating glucagon and liver glucose production [45, 46] while improving liver insulin sensitivity in mice [45, 47]. With these points in mind, the purpose of the current investigation was to examine if fenofibrate, a widely prescribed lipid‐lowering drug [48] and peroxisome proliferator‐activated receptor α (PPARα) agonist which induces FGF21 [49, 50], would protect against acute OLZ‐induced metabolic dysregulation. We hypothesized that prior treatment with fenofibrate would protect against OLZ‐induced increases in serum glucagon and the development of hyperglycemia and lipidemia in an FGF21 dependent manner.

## 2 Materials and Methods

### 2.1 Animal Care

All protocols were approved by the University of British Columbia Animal Care Committee (protocol #A24‐0018) and followed Canadian Council on Animal Care guidelines. Ten‐ to twelve‐week‐old male C57BL/6J mice were purchased from Jackson Laboratories (Bar Harbor, ME, strain# 000664) and group‐housed. Male mice were studied as female mice have previously been shown to be protected against acute olanzapine‐induced hyperglycemia [13]. Rooms were kept at an ambient temperature of 22°C and a 12:12 h light: dark cycle. Mice were acclimated to the facility for a minimum of 1 week before experiments and given ad libitum access to water and standard rodent chow (Teklad, 2918). Whole body *FGF21* ^−/−^ mice were purchased from Jackson Laboratories (Bar Harbor, ME, strain# 033846) and crossed with C57BL/6J mice. *FGF21* ^+/−^ mice were backcrossed to C57BL/6J mice for three generations, and *FGF21* ^−/−^ and wild‐type (WT) littermates were used for experiments. Mice were genotyped using primer sets described previously [51]. Fenofibrate was compounded (0.2% w/w) into a 10% low‐fat diet (Research Diets, D25022011) with sucralose (30 mg/kg diet) added to mask the taste. A low‐fat diet was used to control for weight gain, as pre‐existing obesity exacerbates the effects of OLZ [52]. The control diet was identical but did not contain fenofibrate (Research Diets, D25022010). Pair‐fed mice were given the same amount of food as consumed by the fenofibrate‐treated mice. Mice were provided their respective diets for 7 days prior to the OLZ challenge, and food intake and body weights were measured daily.

### 2.2 Fenofibrate Gavage

Fenofibrate (Cayman, 10 005 368) was dissolved in dimethyl sulfoxide (Sigma‐Aldrich, D5879) to create a stock solution (300 mg/mL) and then 1 mL of solubilized fenofibrate was diluted into 9 mL of 100% corn oil. Mice were gavaged with 300 mg/kg bw fenofibrate, which coincides with the average drug intake/day in diet form, or vehicle (DMSO, Corn Oil). Drug and vehicle solutions were prepared immediately before use. Two hours following fenofibrate gavage, mice were subjected to an OLZ tolerance test.

### 2.3 Metabolic Phenotyping

Indirect calorimetry was performed using the Promethion Metabolic Screening System (Promethion High‐Definition Multiplexed Respirometry System for Mice, Sable Systems International, NV, USA). Body composition was measured with the EchoMRI body composition analyzer (EchoMRI, Houston TX) prior to metabolic caging. Mice were single housed for the duration of the experiment. Mice first underwent a 24‐h baseline acclimation, then metabolic measurements were recorded and analyzed for the subsequent 24‐h. The olanzapine tolerance test was conducted at ~09:00 and measurements were analyzed 2 h prior and post OLZ injection. Respirometry values were determined every 5 min; the dwell time for each cage was 30 s, with baseline cage sampling frequency of 30 s occurring every four cages. The respiratory exchange ratio (RER) was calculated as the ratio of VCO_2_ to VO_2_. Food intake and body mass were recorded continuously by gravimetric measurements within the cages. Locomotion was determined according to *x*, *y* beam breaks within a grid of infrared sensors. Energy expenditure was calculated using the Weir equation (energy expenditure = 3.941 kcal/L × VO_2_ + 1.106 kcal/L × VCO_2_) [53].

### 2.4 Olanzapine Tolerance Test

Olanzapine (Cayman, 11 937) was dissolved in dimethyl sulfoxide (Sigma‐Aldrich, D5879) to create a stock solution (10 mg/mL). A 500 μL of solubilized olanzapine was diluted into 9.5 mL of a Kolliphor EL (Sigma‐Aldrich, C5135)/saline solution (0.5 mL/9 mL). Mice were injected intraperitoneally with olanzapine (5 mg/kg) or vehicle (DMSO, Kolliphor EL, saline) at the beginning of the light cycle (∼09:00), which coincides with clinical recommendations for drug administration before bedtime [54]. Drug and vehicle solutions were prepared immediately before use. Our lab [26, 28, 52] and others [19] have previously used 5 mg/kg olanzapine, as it mimics human dosing requirements based on dopamine‐binding occupancy [55]. Blood glucose was measured in mice at baseline and at 15, 30, 60, 90, and 120 min after drug administration with a FreeStyle Lite glucometer (Freestyle Lite; Abbott Laboratories, IL, USA) sampling blood from a distal tail snip.

### 2.5 Tissue Collection

Mice were anesthetized with sodium pentobarbital (5 mg/100 g bw) and were euthanized by exsanguination. Cardiac blood was collected with 25‐gauge needles (BD PrecisionGlide, 305125) and allowed to clot at room temperature for ~20 min then placed on ice. Serum was harvested by centrifuging blood (5000× *g*, 10 min), then collecting and storing the supernatant at −80°C until further analysis. Livers and inguinal white adipose tissue were dissected and then snap‐frozen in liquid nitrogen and stored at −80°C until further analysis.

### 2.6 Metabolite and Hormone Analysis

All metabolite and hormone assays were analyzed using a Versamax Tunable Microplate Reader and SoftMax Pro Software (Molecular Devices). Serum FGF21 (Sigma‐Aldrich, EZRMFGF21), insulin (10‐1247‐01; Mercodia), and glucagon (10‐1281‐01; Mercodia) were measured with commercially available enzyme‐linked immunosorbent assay (ELISA) kits. Serum NEFA (CA97000‐012; Wako Chemicals), serum beta hydroxybutyrate (BHB) (700190; Cayman Chemicals), and serum and liver triglycerides (10010303; Cayman Chemicals) were measured using commercially available colorimetric assay kits. All assays were performed and analyzed according to the manufacturer's protocol.

### 2.7 Immunoblotting

Inguinal white adipose tissue was homogenized in cell lysis buffer (FNN0021; ThermoFisher) supplemented with a protease/phosphatase inhibitor cocktail (78440; ThermoFisher). Homogenates were centrifuged at 10 000× *g* for 5 min, and the infranatant was collected. Protein concentration was determined by a bicinchoninic acid protein assay (23225; ThermoFisher), and samples for Western blot were prepared by adding 6× Laemmli SDS sample buffer (J61337.AD; ThermoFisher), and then heated to 95°C for 8 min. Samples were then cooled to room temperature and stored at −20°C. A 10 μg of protein was loaded onto an SDS‐PAGE gel (1610172; Bio‐rad) and ran for 90 min at 120 V. Proteins were then transferred (100 V, 60 min, 4°C) to a nitrocellulose membrane (GE10600002; Sigma Aldrich) and then stained with Ponceau S (Sigma‐Aldrich, P7170). Membranes were blocked for 1 h in 5% bovine serum albumin (BSA) (9998S, Cell Signaling) dissolved in 1× Tris‐buffered saline 0.1% Tween (TBS‐T) at room temperature with gentle rocking. The following primary antibodies were diluted 1:1000 in 5% BSA in 1× TBS‐T: phospho‐hormone sensitive lipase (HSL) (Ser660) (45804; Cell Signaling), HSL (4107; Cell Signaling), adipose triglyceride lipase (ATGL) (2138; Cell Signaling), and then incubated for 16 h at 4°C on a rocker. Membranes were washed and then incubated with secondary HRP‐linked anti‐rabbit IgG (7074; Cell Signaling) diluted 1:10 000 in 2% BSA in TBS‐T for 1 h at room temperature. ECL substrate (1705061; Bio‐Rad) was added briefly, and bands were detected using a ChemiDoc Imaging System (12003153; Bio‐Rad). Bands were analyzed by densitometry using AlphaView v 3.3.0 (ProteinSimple, CA, USA) and normalized to total protein detected by ponceau S.

### 2.8 Statistical Analysis

Statistical tests were completed using GraphPad Prism v.10.1.1 (GraphPad Software, CA, USA). Data were analyzed by unpaired, two‐tailed *T*‐tests or two‐way ANOVA, followed by Tukey's *post hoc* analysis if there was a significant interaction, in which case the *p*‐value of group comparisons are shown. Potential outliers were assessed using Grubbs' test. Regression analyses were conducted in R (v4.3.3), homogeneity of regression slopes was tested prior to Type III sums of squares analysis within RStudio v2024.12.0 (Posit Software, MA, USA). Metabolic phenotyping data were processed using CalR [56].

## 3 Results

### 3.1 Fenofibrate Protects Against Olanzapine‐Induced Hyperglycemia

**FIGURE 1:**
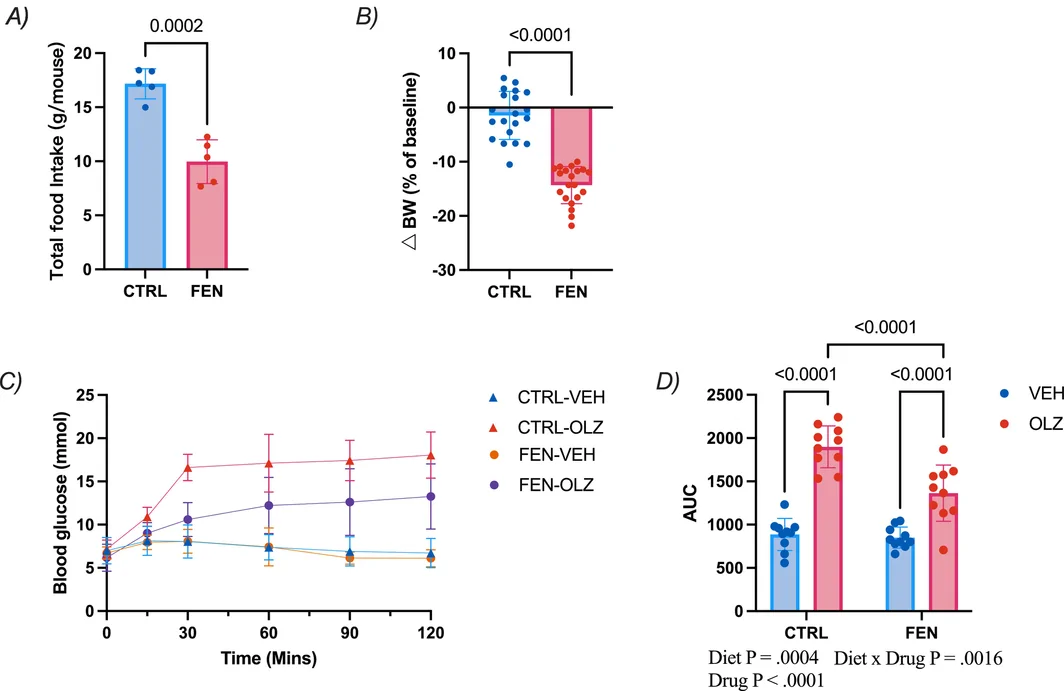
Fenofibrate protects against olanzapine‐induced hyperglycemia. Male mice (*n* = 10) were fed a diet of fenofibrate (0.2% w/w) (FEN) or a control diet (CTRL) for 7 days and food intake (A) and changes in body weight (B) were measured. Mice were then given an intraperitoneal injection of olanzapine (5 mg/kg bw, IP) or vehicle, and (C) blood glucose was measured for 2 h and (D) the area under curve was calculated. Data are presented as mean ± SD for A–D. Food intake and % change body weight were analyzed by two‐tailed unpaired *t*‐test and AUC was analyzed by two‐way ANOVA. When a significant interaction was detected, a Tukey post hoc analysis was completed.

Mice were treated for 7 days with fenofibrate (FEN; 0.2% w/w) or control diet (CTRL). FEN‐treated mice consumed less food (*p* = 0.0002) (Figure 1 A) and lost more weight (*p* < 0.0001) (Figure 1 B) compared to mice given CTRL. As expected, serum FGF21 concentrations were higher in fenofibrate‐treated mice compared to controls (control 791 ± 397, fenofibrate 3667 ± 1222 pg/mL, *p* < 0.0001). Following the treatment period, mice were challenged with OLZ. OLZ increased blood glucose area under the curve (AUC) in both diet groups (*p* < 0.0001) (Figure 1 C,D); however, the hyperglycemic effect of OLZ was blunted in FEN‐treated mice. Previous work from our group has shown that the OLZ‐induced hyperglycemic response is dependent on body weight [52]. Therefore, we analyzed blood glucose AUC by ANCOVA with diet and drug as fixed factors and body weight as a covariate. Body weight exerted a significant covariate effect on blood glucose (*p* = 0.040). After adjustment for body weight, OLZ had a significant main effect to increase blood glucose (*p* < 0.0001). Although there was no main effect of diet (*p* = 0.466), a significant diet × drug interaction was detected (*p* = 0.0053) whereby fenofibrate attenuated the OLZ‐induced increase in blood glucose. Therefore, the persistence of a significant diet × drug interaction after adjustment for body weight suggests that fenofibrate's protective effect is not fully explained by body weight reduction alone.

### 3.2 Calorie Restriction Protects Against OLZ‐Induced Hyperglycemia

**FIGURE 2:**
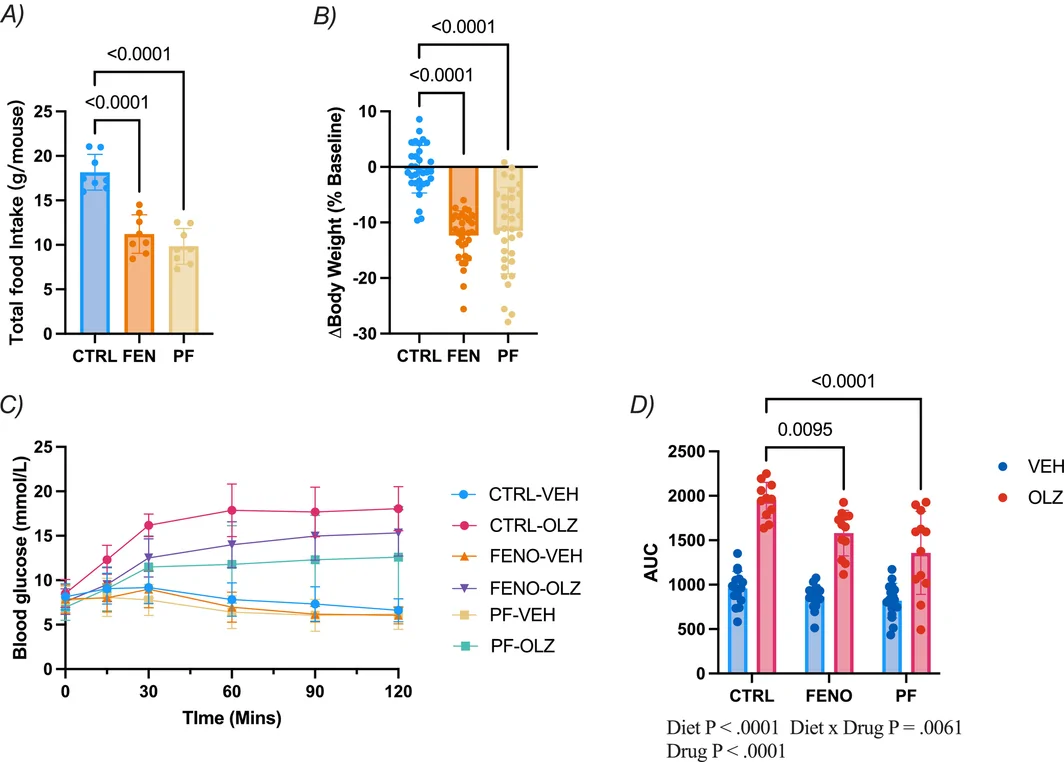
Calorie restriction protects against olanzapine‐induced hyperglycemia. Male mice (*n* = 16 in VEH, *n* = 12 in OLZ) were fed a diet of fenofibrate (0.2% w/w) (FEN), a control diet (CTRL), or a control diet pair‐fed to the fenofibrate group (PF) for 7 days. Food intake (A) and changes in body weight (B) were determined. Mice were then given an intraperitoneal injection of olanzapine (5 mg/kg bw, IP) or vehicle, and (C) blood glucose was measured for 2 h and (D) the area under curve was calculated. Data are presented as mean ± SD. Food intake and changes in body weight were analyzed by one way ANOVA whereas glucose AUC data were analyzed by two‐way ANOVA. When a significant interaction was detected, a Tukey post hoc analysis was completed.

To discern the effects of weight loss from the effects of fenofibrate, we compared the 7‐day fenofibrate diet (0.2% w/w) treatment group to a matched pair‐fed (PF) group. PF mice were provided the same amount of calories as the FEN group. Mice in the FEN and PF groups ate less food (*p* < 0.0001) (Figure 2A) and lost weight (*p* < 0.0001) (Figure 2B) compared to CTRL mice. There was a main effect of OLZ (*p* < 0.0001) to increase blood glucose AUC (Figure 2C,D), and this effect was attenuated in the FEN (*p* = 0.0095) and PF (*p* < 0.0001) groups.

**FIGURE 3:**
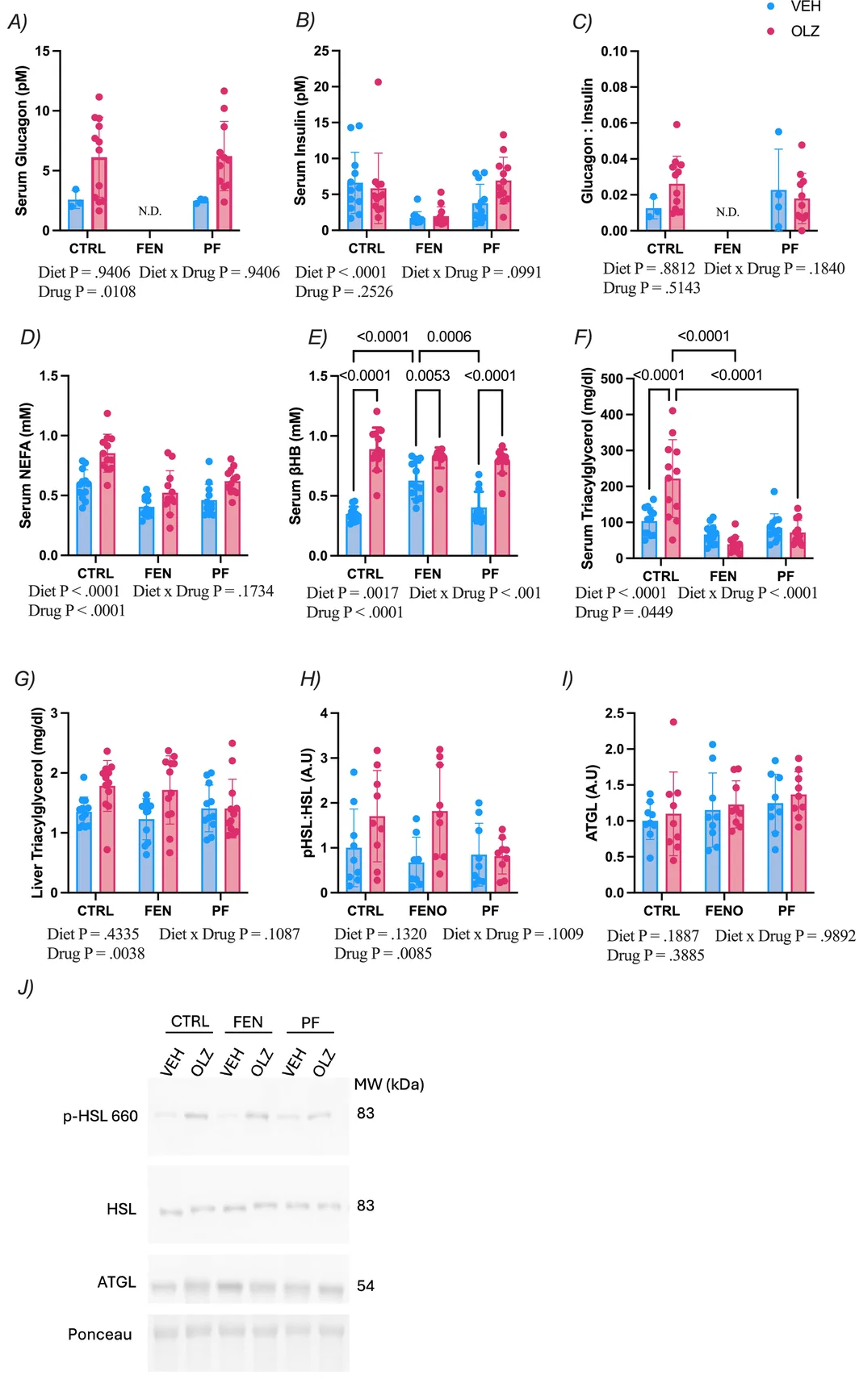
Fenofibrate and pair‐feeding impact olanzapine‐induced alterations in glucoregulatory and lipid homeostasis markers differently. Following 1 week of control diet (CTRL), fenofibrate (FEN), and pair‐feeding (PF) mice were challenged with olanzapine (5 mg/kg bw, IP). (A) glucagon, (B) insulin, (C) the ratio of glucagon to insulin, (D) non‐esterified fatty acids (NEFA), (E) β‐hydroxybutyrate (βHB), and (F) triacylglycerol were measured. (G) Liver triacylglycerol content was quantified and normalized to liver wet weight. A Western blot was performed to determine protein content of (H) the phosphorylation of hormone‐sensitive lipase (HSL) on serine 660 expressed relative to total HSL, (I) and total adipose triglyceride lipase (ATGL) in inguinal white adipose tissue. (J) representative images are shown. Group values that were below detection are labeled as not detected (N.D.) and excluded from statistical analysis. All data are presented as mean ± SD. Differences between groups were analyzed by two‐way ANOVA. When a significant interaction was detected, a Tukey post hoc analysis was completed.

We have previously shown that serum glucagon concentrations increase with OLZ treatment, while insulin concentrations remain unchanged [13, 26, 28]. In the present study we have replicated these findings, demonstrating that olanzapine increased serum glucagon (Figure 3A). There were main effects of OLZ (*p* = 0.0108) to increase serum glucagon in CTRL and PF mice, while glucagon concentrations were below the limit of detection in FEN‐treated mice. There was a main effect of diet on serum insulin, with insulin being reduced in FEN‐treated mice (*p* < 0.0001) (Figure 3B). The ratio of glucagon to insulin, a measure that is linked to dysglycemia [57, 58], could not be reliably quantified for FEN‐treated mice as glucagon levels were below detection. After exclusion of this group, there were no significant main effects or interaction in glucagon: insulin ratios between the PF and CTRL groups (Figure 3C).

We next investigated if fenofibrate or pair feeding could offset acute olanzapine‐induced disturbances in lipid metabolism. There was an effect of OLZ to increase serum non‐esterified fatty acids (NEFAs) (*p* < 0.0001), and an effect of diet to lower NEFAs in the FEN and PF groups (*p* < 0.0001) (Figure 3D). OLZ increased circulating concentrations of β‐hydroxybutyrate (BHB) in all three groups, while BHB in vehicle‐treated mice was greater in the fenofibrate compared to the control (*p* < 0.0001) and PF (*p* = 0.0006) mice (Figure 3E). OLZ treatment increased serum triglycerides in CTRL mice (*p* < 0.0001), and this effect was prevented with fenofibrate treatment (*p* = 0.8101) or pair feeding (*p* = 0.9898) (Figure 3F). Serum triacylglycerol (TAG) concentrations under OLZ‐stimulated conditions were greater in control compared to fenofibrate (*p* < 0.0001) and PF (*p* < 0.0001) mice. In the liver, olanzapine increased TAG accumulation (*p* = 0.0038) (Figure 3G). Lastly, in inguinal white adipose, there were main effects of olanzapine to increase phosphorylated HSL at ser 660 relative to total HSL (*p* = 0.0085), but not ATGL (*p* = 0.3885) (Figure 3H–J). Taken together, our findings demonstrate that fenofibrate protects against OLZ‐induced hyperglycemia and attenuates OLZ‐induced increases in circulating triglycerides. These effects were recapitulated by pair‐feeding, suggesting a negative energy balance contributes to these metabolic benefits.

### 3.3 Acute Gavage of Fenofibrate Does Not Attenuate Olanzapine‐Induced Hyperglycemia

**FIGURE 4:**
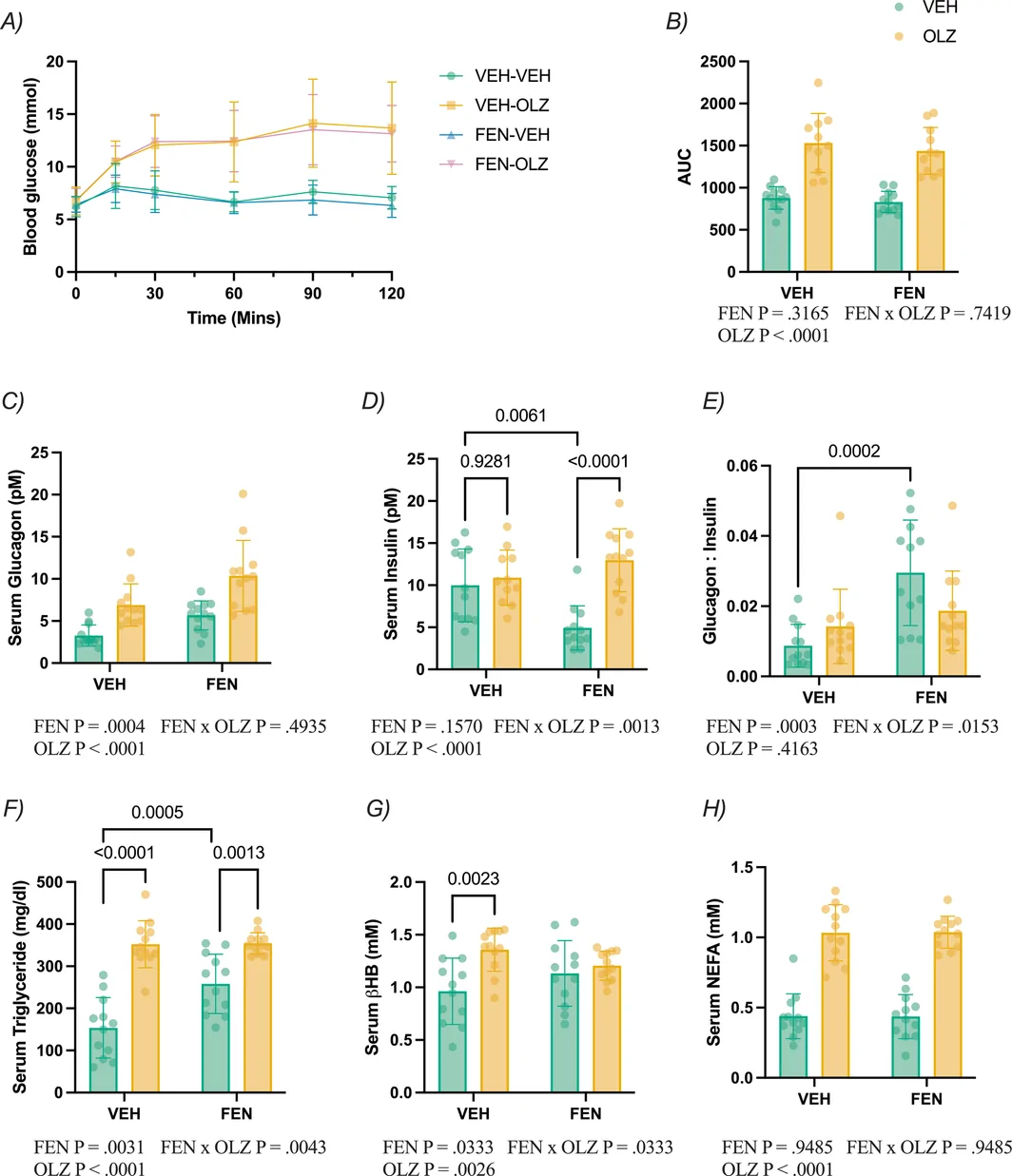
Acute gavage of fenofibrate does not attenuate olanzapine‐induced hyperglycemia. Male mice (*n* = 12) were gavaged with fenofibrate (300 mg/kg bw) or vehicle, followed by an intraperitoneal injection of olanzapine (5 mg/kg bw, IP) or vehicle. (A) Blood glucose was measured for 2 h and (B) area under curve was calculated. Serum (C) glucagon, (D) insulin, (E) the glucagon insulin ratio, (F) triglycerides, (G) beta‐hydroxybutyrate, (H) and non‐esterified fatty acids (NEFA) were measured. All data are presented as mean ± SD. Differences between groups were analyzed by two‐way ANOVA. When a significant interaction was detected, a Tukey post hoc analysis was completed.

To examine the effects of fenofibrate independent of weight loss, we acutely gavaged mice with fenofibrate and 2 h later treated them with olanzapine. Two hours following fenofibrate gavage serum FGF21 concentrations (vehicle 592 ± 399, fenofibrate 3766 ± 972 pg/mL, *p* = 0.0012) were increased to a similar extent as was shown with 1 week of fenofibrate feeding. There was a main effect of OLZ to increase blood glucose AUC (*p* < 0.0001) (Figure 4A,B) and this was not impacted by prior treatment with fenofibrate. There were main effects of both OLZ (*p* < 0.0001) and fenofibrate (*p* = 0.0004) to increase serum glucagon (Figure 4C). In fenofibrate‐treated mice, serum insulin was increased with OLZ treatment compared to vehicle (*p* < 0.0001) (Figure 4D). There was a main effect of fenofibrate to increase the glucagon to insulin ratio (*p* = 0.0003) (Figure 4E) and main effects of OLZ to increase serum triglycerides (*p* < 0.0001) (Figure 4F), BHB (*p* = 0.0026) (Figure 4G), and NEFAs (*p* < 0.0001) (Figure 4H). Together, these findings demonstrate that acute treatment with fenofibrate is not sufficient to protect against acute OLZ‐induced hyperglycemia and hyperlipidemia.

### 3.4 The Protective Effects of Fenofibrate Against Olanzapine‐Induced Hyperglycemia Are Partially FGF21 Dependent

**FIGURE 5:**
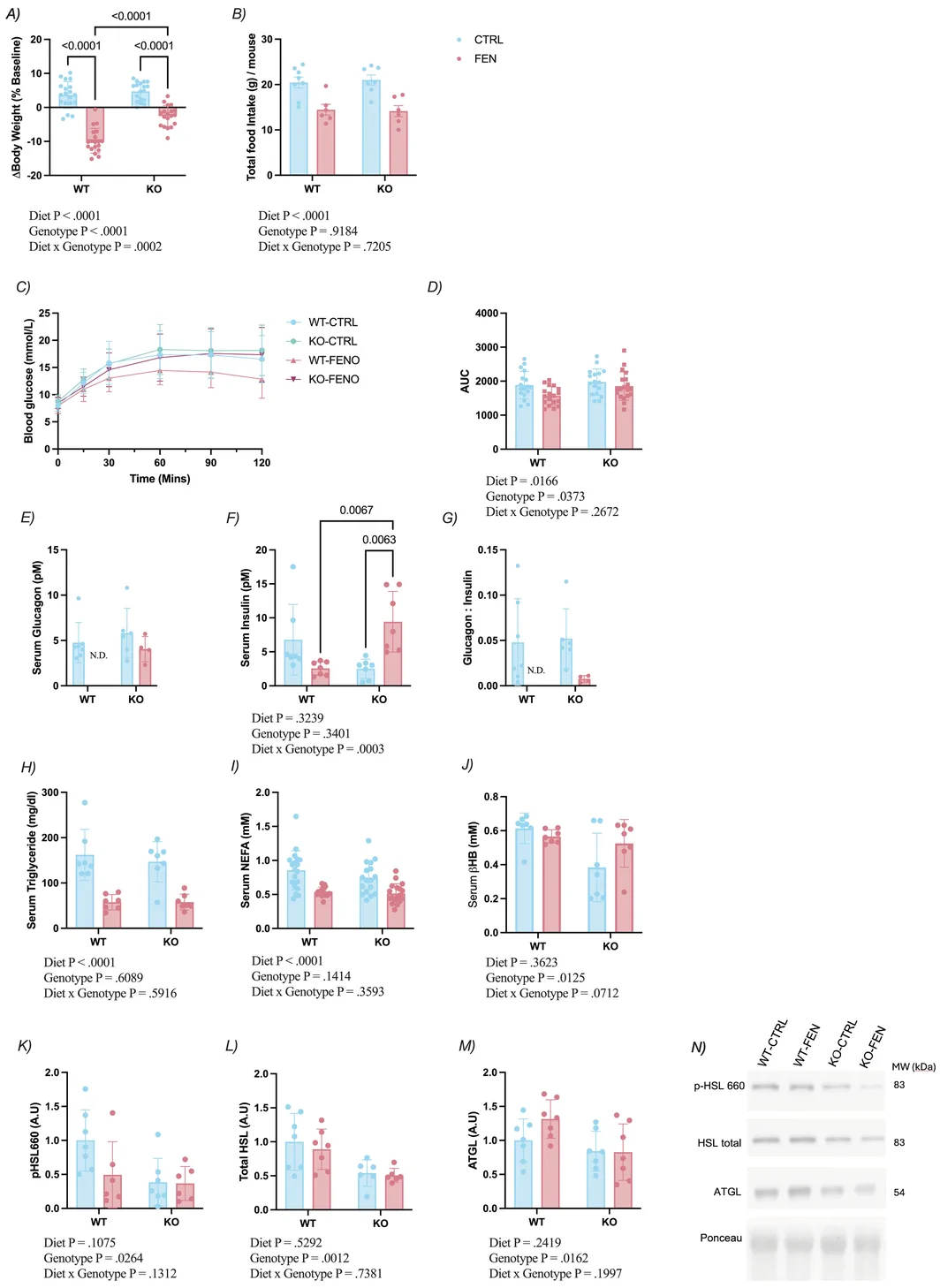
The protective effects of fenofibrate against olanzapine‐induced hyperglycemia are partially FGF21 dependent. *FGF21* ^−/−^ and littermate WT male mice (*n* ~ 18) were fed a diet of 0.2% fenofibrate (w/w) (FEN) or control diet (CTRL) for 7 days and food intake (A) and (B) changes in body weight were measured. Mice were then treated with olanzapine (5 mg/kg bw, IP) and (C) blood glucose measured for 2 h and (D) area under curve calculated. (E) Glucagon, (F) insulin, and (G) the ratio of glucagon to insulin (*n* = 7). (H) triacylglycerol (I) *n*on‐esterified fatty acids (NEFA), and (J) beta‐hydoxybutyratewere (βHB) were measured. A Western blot was performed to determine protein content of (K) the phosphorylation of hormone‐sensitive lipase (HSL) on serine 660, (L) total HSL, (M) and total adipose triglyceride lipase (ATGL) in inguinal white adipose tissue, with (N) representative images shown. Group values that were below detection are labeled as not detected (N.D.) and excluded from statistical analysis. All data are presented as mean ± SD. Differences between groups were analyzed by two‐way ANOVA.

To determine whether FGF21 is required for fenofibrate's protective effect against olanzapine‐induced hyperglycemia, we repeated the 7‐day dietary fenofibrate treatment in *FGF21* ^*−/−*^ and littermate WT mice and then challenged the mice with OLZ. WT (*p* < 0.0001) and KO (*p* < 0.0001) mice fed fenofibrate both lost body weight, though weight loss was diminished in FGF21 deficient mice (*p* < 0.0001) (Figure 5A). There was a main effect of fenofibrate to reduce food intake (*p* < 0.0001), and this effect did not differ between genotypes (Figure 5B). Following an OLZ challenge, there were main effects of genotype (*p* = 0.037) to increase glucose AUC and main effects of diet (*p* = 0.017) to decrease glucose AUC (Figure 5C,D). When examining the effects of fenofibrate in each genotype, fenofibrate significantly reduced glucose AUC in WT (*p* = 0.014), but not *FGF21* ^*−/−*^ mice (*p* = 0.344). Fenofibrate lowered serum glucagon to below the limit of detection in WT animals, and this effect was attenuated in *FGF21* ^*−/−*^ mice (Figure 5E). Serum insulin was greater in *FGF21* ^−/−^ mice treated with fenofibrate compared to WT fenofibrate mice (*p* = 0.0067) (Figure 5F). Due to glucagon being below the limit of detection in fenofibrate‐treated WT mice, the glucagon: insulin ratio could not be quantified for this group. In fenofibrate‐treated *FGF21* ^−/−^ mice, the glucagon: insulin ratio remained quantifiable, and was lower than in control‐treated FGF21‐deficient mice (Figure 5G). Fenofibrate lowered serum triacylglycerol (*p* < 0.0001) and serum NEFAs (*p* < 0.0001) (Figure 5H,I). Additionally, there was a main effect of genotype to reduce serum BHB (*p* = 0.0125) (Figure 5J). Lastly, in inguinal white adipose, there were main effects of genotype to reduce the protein content of phosphorylated HSL ser 660 (*p* = 0.1075), HSL (*p* = 0.0012), and ATGL (*p* = 0.0162) (Figure 5K–N). Collectively these findings provide evidence that fenofibrate‐induced weight loss, and reductions in olanzapine‐induced hyperglycemia and increases in glucagon, but not the development of hyperlipidemia, are partially dependent upon FGF21.

### 3.5 FGF21 Is Required for OLZ‐Induced Reductions in RER

**FIGURE 6:**
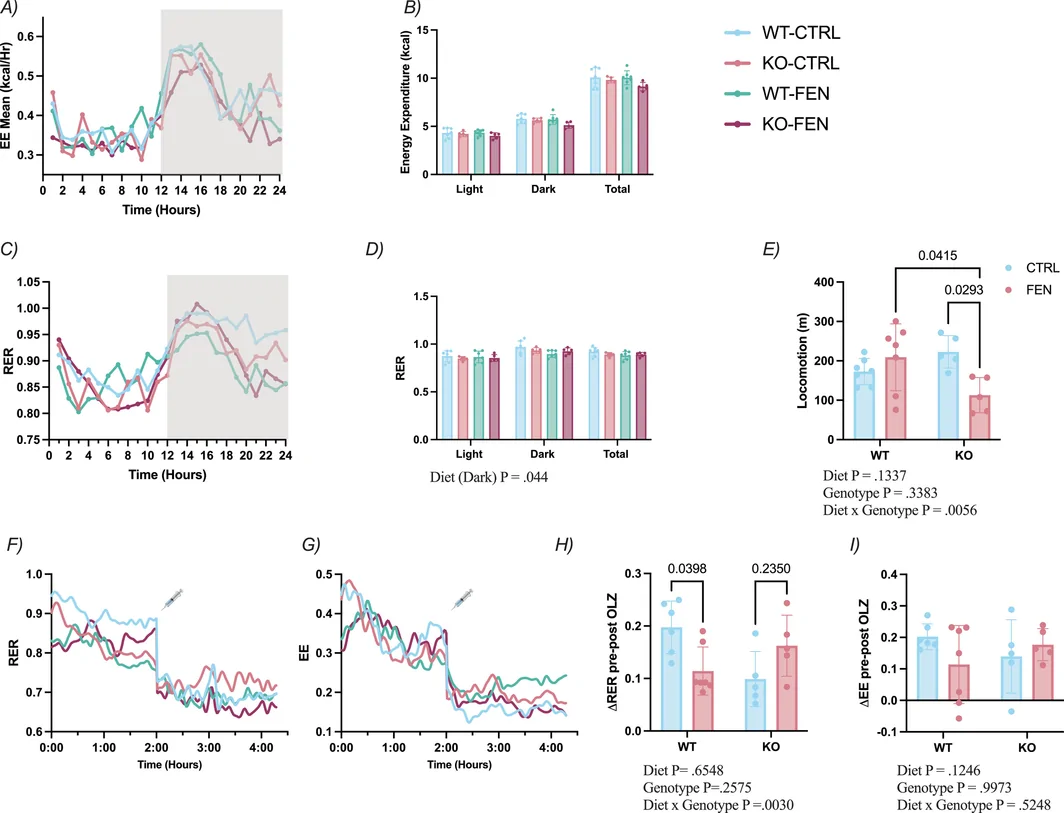
FGF21 is required for OLZ‐induced reductions in RER. FGF21^−/−^ (*n* = 5) and littermate WT male mice (*n* = 7) were fed a 0.2% fenofibrate (w/w) or control diet (CTRL) for 7 days. Mice were placed in metabolic caging on day 5, and (A) 24‐h energy expenditure (EE) and (B) EE in each light cycle were measured. (C) 24‐h respiratory exchange ratio (RER) and (D) average RER in each light cycle measured. Cumulative locomotion is shown in (E). On day 7, mice were injected with olanzapine (5 mg/kg bw, IP), and RER (F) and EE (G) were measured over the subsequent 2‐h period, and the change RER (H) and EE (I) after olanzapine treatment was calculated. Data are presented as the mean for A, C. Data are presented as mean ± SD for B, D, E, H, I. Differences between groups were analyzed by two‐way ANOVA.

To understand how fenofibrate could be impacting energy expenditure and substrate oxidation in response to OLZ, *FGF21* ^−/−^ and WT mice underwent indirect calorimetry following treatment. Energy expenditure (EE) was measured over a 24‐h period (Figure 6A), and total EE per light cycle was calculated (Figure 6B). Lean mass is a primary determinant of EE [59] and therefore average EE was analyzed by ANCOVA using lean mass as a covariate. Homogeneity of regression slopes was confirmed. Adjusted EE revealed a significant diet × genotype interaction (*p* = 0.0210), with FGF21‐deficient mice treated with fenofibrate displaying reduced EE compared to controls. The respiratory exchange ratio (RER) was measured over a 24‐h period (Figure 6C), and the average RER per light cycle was calculated (Figure 6D). We observed a main effect of fenofibrate to lower RER during the dark cycle (*p* = 0.044). *FGF21* ^−/−^ mice treated with fenofibrate traveled less distance compared to controls (Figure 6E).

Following the 24‐h metabolic caging analysis, animals were treated with OLZ and changes in whole‐body substrate oxidation and energy expenditure were measured (Figure 6F,G). OLZ‐induced changes in RER depended on genotype and diet, (Figure 6H) as a significant interaction was observed (*p* = 0.0030). Post hoc analysis revealed fenofibrate blunted reductions in RER (i.e., smaller treatment delta) in WT mice (*p* = 0.0398), a relationship that was absent in *FGF21* ^−/−^ animals. There were no significant effects of diet (*p* = 0.1246) or genotype (*p* = 0.9973) on changes in EE (Figure 6I).

## 4 Discussion

PPARα agonists like fenofibrate have been shown to decrease plasma glucose, triglycerides, and insulin [49, 60]. To date, the effect of fibrates on acute AP‐induced perturbations in glucose and lipid metabolism has not been investigated. In this study, we find that dietary fenofibrate treatment reduced food intake, caused weight loss, and protected against olanzapine‐induced hyperglycemia. While covarying for final body weight did not obscure the protective effect of fenofibrate, we found that pair‐feeding mice the same amount of calories as fenofibrate‐treated animals resulted in similar protective effects as fenofibrate against OLZ‐induced hyperglycemia. Along a similar line, despite causing large increases in circulating concentrations of FGF21, an acute oral gavage of fenofibrate did not protect against OLZ‐induced increases in blood glucose. Collectively, these findings provide evidence that the protective effects of fenofibrate against OLZ‐induced hyperglycemia are likely secondary to reductions in body weight.

The protective effects of fenofibrate were likely mediated, at least in part, by FGF21. In support of this, there were main effects of genotype to increase the glucose response to OLZ, while fenofibrate reduced OLZ‐induced hyperglycemia in WT but not *FGF21* ^*−/−*^ mice. Similarily, serum glucagon was reduced to a greater degree in WT compared to *FGF21* ^*−/−*^ mice. Together, this provides evidence that a portion of the protective effect of fenofibrate against OLZ‐induced increases in blood glucose is mediated by FGF21.

Increases in circulating glucagon play a central role in the development of hyperglycemia following OLZ treatment. In this regard, many interventions which protect against OLZ‐induced hyperglycemia, such as fasting [29], ketogenic diets [29], and exercise [17], also blunt increases in glucagon. Importantly, OLZ‐induced excursions in blood glucose are absent in glucagon‐receptor‐deficient mice [26]. Prior work has suggested that FGF21 can suppress serum glucagon [45, 46], and in this study, we demonstrate that fenofibrate dampened the effects of OLZ on serum glucagon in an FGF21‐dependent manner. Interestingly, the effects of fenofibrate to attenuate OLZ‐induced increases in glucagon were only apparent if weight loss occurred. In support of this, a single oral gavage of fenofibrate, despite increasing serum FGF21 concentrations to a similar level as with long‐term dietary fenofibrate, did not protect against OLZ‐induced excursions in blood glucose. Interestingly, despite PF mice losing the same amount of weight as fenofibrate‐treated mice, the glucagon response to OLZ was intact. This would suggest distinct mechanisms of action between fenofibrate treatment and caloric restriction in regard to protection against OLZ‐induced excursions in blood glucose.

Increased lipid mobilization and enhanced fat oxidation are well‐documented side effects of acute olanzapine treatment and are thought to be a compensatory response to reductions in carbohydrate utilization [20]. In the current study, we found that OLZ‐induced increases in serum NEFAs, TAGs, and whole‐body fatty acid oxidation (reductions in RER) were prevented by fenofibrate, however, only changes in RER were dependent upon FGF21. These results provide evidence of an uncoupling between acute perturbations in lipid and glucose metabolism. Previous work from our group has shown similar effects, where voluntary wheel running protected against hyperglycemia but not increases in lipids following acute treatment with OLZ [17]. It is somewhat surprising that reductions in serum fatty acids with fenofibrate did not require FGF21, as prior work has shown that FGF21 can suppress lipolysis [61]. Conversely, the mRNA expression [62] and protein content (Figure 5K,L) of HSL and ATGL are reduced in white adipose tissue from *FGF21* ^−/−^ mice.

Prior work has shown that fenofibrate reduces food intake [63, 64] and prevents high‐fat diet‐induced weight gain in mice [49, 65]. Although it is not widely investigated as a weight loss agent in humans, one study reported greater reductions in BMI and percent body fat in patients with obesity treated with metformin and fenofibrate compared to metformin alone [66]. In the current study, we found that fenofibrate, in the setting of a low‐fat diet, caused weight loss in a manner that was partially dependent upon FGF21. The dependency on FGF21 was not explained by energy intake, as fenofibrate‐induced reductions in food intake were similar in both WT and *FGF21* ^−/−^ mice. Rather, WT fenofibrate‐treated mice appeared to maintain lean‐mass‐adjusted energy expenditure, while KO fenofibrate mice had relatively lower adjusted energy expenditure.

The current findings provide evidence that the PPARα agonist, fenofibrate, alleviates some of the acute metabolic perturbations induced by antipsychotics such as olanzapine. Similarly, previous studies showed co‐treatment with fenofibrate also appears to prevent increases in blood glucose and serum triglycerides following longer term treatment with the antipsychotic clozapine in mice [67]. In addition to protecting against the metabolic complications of antipsychotics, there is evidence to suggest that targeting PPARα could alleviate symptoms of schizophrenia. In this regard, mutations in *PPARA* have been identified as risk variants for schizophrenia in Japanese patients, whereas *PPARα* ^−/−^ mice exhibit schizophrenia‐relevant phenotypes [68]. Interestingly, fenofibrate treatment in mice mitigated schizophrenia symptoms such as dendritic spine loss induced by phencyclidine and reduced hyper‐locomotion induced by MK‐801, drugs which competitively bind the NMDA receptor in the brain [68]. These findings provide evidence that fenofibrate could be used to target anatomical and behavioral symptoms of schizophrenia.

In summary, the results of the current study demonstrate that fenofibrate could be a clinically relevant treatment in patients living with schizophrenia as it can confer protections against hyperglycemia and hyperlipidemia following treatment with the antipsychotic OLZ. Protection against perturbations in glucose, but not lipid metabolism, was dependent upon FGF21. Given the reported effects of fenofibrate on reducing schizophrenia symptomology in rodent models [68], fenofibrate could be an attractive candidate to be paired with antipsychotics in the treatment of schizophrenia.

## Author Contributions

Michael Akcan, Margaret Hahn, and David C. Wright designed the experiments. Michael Akcan, Stewart Jeromson, Bradley J. Baranowski, Annalaura Bellucci, Serena Trang, Katelyn Eisner, Ailish Babicki‐Moore, Hadil Alfares, Meagan Arbeau, and Kyle Medak performed the experiments. Michael Akcan, Stewart Jeromson, and David C. Wright analyzed the data. Michael Akcan and David C. Wright wrote the paper. All authors edited the manuscript.

## Funding

This work was supported by Canadian Institutes of Health Research (CIHR), PJT‐191735.

## Conflicts of Interest

M.K.H. has received consultant fees from Alkermes Inc., Merck, and Eli Lilly. Other authors declare no conflicts of interest.

## Data Availability

The data that support the findings of this study are available in the Materials and Methods, and Results sections of this article.
